# Does perceived motor competence and health-related physical fitness mediate the relationship between actual motor competence and physical activity in middle and late childhood?

**DOI:** 10.3389/fpsyg.2026.1686950

**Published:** 2026-02-19

**Authors:** Gang Sun, Renke He, Jiaying Zhang, Yi Chen, Wenxin Li, Zan Huang, Yulan Zhou

**Affiliations:** 1 College of Physical Education and Health Sciences, Zhejiang Normal University, Jinhua, Zhejiang, China; 2 Shuguang Primary School, Jinhua, Zhejiang, China; 3 Rongguang School, Jinhua, Zhejiang, China; 4 School of Physical Education and Sport Science, Fujian Normal University, Qishan Campus of Normal University, Fuzhou, Fujian, China

**Keywords:** actual motor competence, childhood, health-related physical fitness, perceived motor competence, physical activity

## Abstract

**Background:**

Limited evidence exists regarding the mediating roles of perceived motor competence (PMC) and health-related physical fitness in the relationship between actual motor competence (AMC) and moderate-to-vigorous physical activity (MVPA), particularly across middle and late childhood.

**Objectives:**

This cross-sectional study examined whether PMC and health-related physical fitness mediate the AMC-MVPA relationship in children across middle and late childhood, while exploring differences between these two developmental periods.

**Methods:**

A total of 578 Chinese children, comprising 273 children in middle childhood (mean age = 8.4 ± 0.52 years; 52.3% girls) and 305 in late childhood (mean age = 11.6 ± 0.68 years; 50.5% girls), participated in this study. AMC was evaluated using the Test of Gross Motor Development-3, PMC was assessed with the Pictorial Scale of Perceived Competence and Social Acceptance and the Self-Perception Profile for Children, MVPA was measured via accelerometers, and health-related physical fitness was determined through body mass index, vital capacity, 50-m dash, sit-and-reach test, and 1-min rope-skipping test. Data were analyzed using structural equation modeling.

**Results:**

For middle childhood (6–9 years), AMC showed direct effects on PMC (β = 0.43, *p* < 0.001), MVPA (β = 0.25, *p* < 0.001), and health-related physical fitness (β = 0.53, *p* < 0.001), with significant indirect effects on MVPA through both PMC (β = 0.04, *p* < 0.001) and physical fitness (β = 0.08, *p* < 0.001), accounting for 38.9% of MVPA variance. In late childhood (10–12 years), AMC directly influenced PMC (β = 0.81, *p* < 0.001) and MVPA (β = 0.45, *p* < 0.001), with an indirect effect through PMC (β = 0.11, *p* < 0.001), explaining 15.6% of MVPA variance.

**Conclusion:**

The mediating pathways linking AMC to MVPA demonstrate a distinct developmental shift. Health-related physical fitness serves as a prominent mediator in middle childhood, yet its influence attenuates in late childhood. In contrast, PMC maintains a stable mediational role across both periods. Interventions designed to promote PA via motor competence must be developmentally tailored: prioritizing fitness enhancement in middle childhood and shifting focus to address evolving psychosocial barriers in later years.

## Introduction

Regular participation in physical activity (PA) is associated with various health outcomes in children, including maintaining a healthy body weight, reducing the risk of diabetes, hypertension, and cardiovascular diseases, and enhancing emotion regulation and stress management (Janssen and Leblanc, 2010; Poitras et al., 2016; Bao et al., 2024). In this regard, the World Health Organization (WHO) recommends that children engage in at least 60 min of moderate-to-vigorous physical activity (MVPA) each day (World Health Organization [WHO], 2020). However, globally, most children do not meet this recommendation (Afthentopoulou et al., 2018; Finger et al., 2018; Guthold et al., 2020). Specifically, in China, only 50.0% of children achieve the suggested MVPA levels (Liu et al., 2022). Identifying the correlates and mechanisms of PA and their interactions is crucial for developing effective strategies to improve children’s PA levels.

Among the key factors believed to influence PA engagement is a child’s actual motor competence (AMC). AMC is defined as the skillful execution of a broad array of motor tasks, as well as the coordination and control necessary for achieving specific movement outcomes (Utesch and Bardid, 2019). It is considered as a prerequisite for children to participate in PA. Studies have shown a positive relationship between AMC and PA, with higher AMC levels correlating with increased PA during childhood (Holfelder and Schott, 2014; Logan et al., 2015). However, the relationship between AMC and PA is likely not merely direct; it is also influenced by psychological and physiological factors. One critical psychological factor is perceived motor competence (PMC), which refers to an individual’s awareness and belief in their ability to execute sports and physical activity-related skills (Harter, 2012). This perception plays a crucial role in determining whether a child will continue to engage in an activity (Eccles and Wigfield, 2002). Studies examining the connection between PMC and PA indicate low to moderate positive correlations (Davison et al., 2006; Barnett et al., 2008). Similarly, from a physiological perspective, health-related physical fitness is a vital component that impacts children’s participation in PA. Higher levels of fitness, including aspects such as cardiorespiratory fitness, muscular strength, muscular endurance, and flexibility, may support sustained engagement in PA over extended periods (Bouchard et al., 2007; Chen et al., 2018).

To synthesize these interrelated concepts, Stodden et al. (2008) introduced a conceptual model. This model posits that PMC and health-related physical fitness are important mediators in the link between AMC and PA in childhood (Stodden et al., 2008). Children’s perceived competence may change based on the outcomes of their skill performance (Johnson et al., 2022). Those with adequate levels of AMC are more likely to persist in sports activities, which helps them develop or maintain various aspects of health-related physical fitness (Cattuzzo et al., 2016). Therefore, AMC can improve children’s PA levels through its effects on PMC and health-related physical fitness. In recent years, empirical studies have tested the mediating pathways proposed in the Stodden model, yielding mixed findings regarding the roles of PMC and health-related physical fitness. For instance, Khodaverdi et al. (2016) found that PMC mediated the relationship between AMC and PA in middle childhood, and Gu et al. (2017) confirmed a similar mediation effect in late childhood. However, this mediating role is not consistently supported. For example, De Meester et al. (2018), studying a sample of children in middle to late childhood, and Crane et al. (2023), focusing on middle childhood, found no significant mediation through PMC. These discrepancies may be attributable to methodological variations across studies, including the use of different tools to assess PMC (e.g., pictorial scales vs. questionnaires) and AMC (e.g., product- vs. process-oriented assessments), as well as differences in the specific age ranges and cultural contexts of the participant samples. A more consistent pattern emerges for health-related physical fitness, with studies by Gu et al. (2021), Jaakkola et al. (2019) identifying its significant mediating role in both middle and late childhood, though the specific fitness components measured also varied.

Despite this growing body of evidence, a critical question remains unaddressed: how do these mediating pathways evolve as children develop? While research has revealed the mediating roles of PMC and health-related physical fitness in the link between AMC and PA during childhood, there has been no investigation into age differences in these mediating effects. With the development of cognitive abilities, children become increasingly capable of accurately evaluating their competence during middle childhood (Robinson et al., 2015). This suggests that the AMC-PMC relationship strength with age, indicating potential age-related differences in the role of PMC as a mediator between AMC and PA (de Witte et al., 2022). In addition, the development of AMC and physical abilities during childhood is influenced by the maturation of physiological structures and systems, as well as the evolution of the sensorimotor apparatus (Lima et al., 2019). This leads to a stronger relationship between AMC and health-related physical fitness in later childhood compared to early childhood, suggesting a possible age-related effect of health-related physical fitness on the mediating role of AMC in PA (Stodden, 2014). To address these gaps, this study aimed to (a) examine whether PMC and health-related physical fitness mediate the AMC-MVPA relationship in middle (6–9 years) and late childhood (10–12 years), and (b) investigate potential age-related differences in these mediating pathways.

## Materials and methods

The study was conducted in accordance with the relevant ethical guidelines and approved by the Ethics Committee of Zhejiang Normal University (No. ZSRT2024210). A convenience sampling methodology was employed to recruit participants from two primary schools in Jinhua City, Zhejiang Province, China. The study population comprised students from all six grade levels (Grades 1–6), aged 6–12 years. Within each grade level, two classes were randomly selected, with class sizes ranging from 25 to 45 students. The initial recruitment phase enrolled 867 eligible participants from 24 classes. After obtaining written informed consent from legal guardians and verbal assent from participants, 797 individuals (representing 91.9% of the initial sample) were formally enrolled prior to data collection. During the data quality assessment phase, 87 participants were excluded due to insufficient accelerometer data, which failed to meet the minimum wear-time criterion of ≥10 h on at least three valid days (including two weekdays and one weekend day). Furthermore, the dataset revealed missing values across critical measures: 60 in objectively assessed actual motor competence, 13 in self-reported perceived motor competence, and 59 in health-related physical fitness assessments. After these exclusions, the final analytical sample included 578 participants with complete data for all primary outcome measures.

### Actual motor competence

The Test of Gross Motor Development-3rd edition (TGMD-3) was employed to assess AMC. It consists of two subscales: (a) locomotor skills, including running, galloping, hopping, skipping, horizontal jumping, and sliding; and (b) ball skills, including two-hand striking of a stationary ball, one-hand forehand striking of a self-bounced ball, one-hand stationary dribbling, two-hand catching, kicking a stationary ball, overhand throwing, and underhand throwing (Ulrich, 2019). To ensure reliability, each skill and its criteria in the TGMD-3 were assessed by two experienced observers. Both observers had completed the TGMD-3 reliability test using video analysis and had extensive experience with live and video-recorded observations. Before each practice trial, the researcher demonstrated each skill once. Following the TGMD-3 manual, two assessment trials were conducted. Children’s performance on each skill was scored as 1 (present) or 0 (absent). The AMC score was calculated by summing the scores of the locomotor and ball skills subscales, with raw test scores used for analysis. The TGMD-3 is known for its good to excellent intra-rater and inter-rater reliability. In this sample, inter-rater reliability was assessed by having both observers independently code the same performances of 578 children, resulting in an inter-rater reliability of 0.85 for the AMC score.

### Perceived motor competence

The perceived physical competence subscale of the Pictorial Scale of Perceived Competence and Social Acceptance (PSPCSA) was used to assess PMC for first- and second-grade children (Harter and Pike, 1984). For third- through sixth-grade children, the athletic competence subscale of the Self-Perception Profile for Children (SPPC) was employed (Harter, 1985). Both subscales have demonstrated good reliability and validity within their target populations (Harter and Pike, 1984; Harter, 1985). Each scale includes six items. During the assessment, children were presented with two types of children (illustrated through pictures or described in sentences) and asked to select the one most like themselves. They then indicated whether their choice was “sort of true” or “really true” for them. Each item was scored on a scale from 1 (low perceived competence) to 4 (high perceived competence). The total PMC score was calculated by summing the six items, with higher scores indicating greater perceived competence. In this study, 578 children were retested on the PSPCSA and SPPC after 14 days, resulting in test-retest reliability scores of 0.85 for the PSPCSA and 0.83 for the SPPC.

### Moderate-to-vigorous physical activity

Moderate-to-vigorous physical activity was assessed using Actigraph wGT3X+ accelerometers, reliable and valid devices that are small and lightweight, making them ideal for use with children (Robusto and Trost, 2012). Children were instructed to wear the accelerometer on their right hip throughout waking hours for seven consecutive days, except during water-based activities (e.g., swimming or bathing). Data were collected as raw accelerations at 30 Hz, processed through standard filtering, and converted into 15-s epochs (Liu et al., 2024). After data collection, accelerometer data were extracted and processed using ActiLife 6.5 software. Non-wear time was defined as any period of at least 60 consecutive minutes with 0 counts per minute recorded by the accelerometer (Troiano et al., 2008). To be eligible for inclusion in the analyses, children were required to have a minimum of 10 h of accelerometer wear time per day on at least 3 days, including two weekdays and one weekend day (Colley et al., 2010). Zhu’s et al. (2013) MVPA threshold (≥2,800 counts per minute) was applied to classify activity levels as moderate-to-vigorous intensity and to quantify time spent in this intensity range (Zhu et al., 2013).

### Health-related physical fitness

Children’s health-related physical fitness in this study was assessed using the 2014 revision of the Chinese National Standard of Physical Fitness for Students (CNSPFS), a testing protocol mandated by the Chinese Ministry of Education (Ministry of Education of the People’s Republic of China, 2014). The CNSPFS includes five to seven assessments: BMI (kg/m^2^), forced vital capacity of the lungs (spirometry), 50-m dash, sit-and-reach test, 1-min rope-skipping, 1-min sit-ups (for students in grades 3–6), and the 50-m × 8 shuttle run (for students in grades 5–6). These assessments evaluate body composition, cardiorespiratory fitness, speed, flexibility, motor coordination, muscle strength, and cardiorespiratory endurance, respectively. To ensure consistency, five tests applicable to all children—BMI, vital capacity, 50-m dash, sit-and-reach test, and 1-min rope-skipping—were selected to assess children’s health-related physical fitness. The specific objectives and administration procedures for each test have been detailed in a prior study (Zhu et al., 2017). The individual test results were converted into percentage scores for specific grades and genders using the CNSPFS scoring method. Then, the average scores of the five test items were calculated to determine an overall health-related physical fitness score.

### Covariates

Children’s gender, parent’s education level, and family socio-economic status (SES) were included as covariates in the statistical analyses due to their relationship with the study variables (Barnett et al., 2009; Morrison et al., 2018). Parents reported their education level, which was categorized into two groups based on the highest education level obtained by either the mother or the father: (a) below university education, and (b) university education. In addition, parents also reported their household income. Based on Zhejiang’s per capita disposable income, the family annual income was categorized as either lower or higher SES. Information on these covariates were obtained from the parent questionnaire.

### Data analysis

Before conducting statistical analyses, missing values and outliers were removed, and the normality of the data was checked. Descriptive statistics characterized the sample, and independent samples *t*-tests examined developmental differences between middle and late childhood groups. Pearson’s correlation coefficients were used to investigate associations among AMC, PMC, MVPA, and health-related physical fitness. The strength of the correlations was interpreted based on Cohen’s criteria, which classified correlations as weak (*r* = 0.10–0.29), moderate (*r* = 0.30–0.49), or high (*r* ≥ 0.50) (Cohen, 2013). To examine the mediating effects, structural equation modeling (SEM) was employed to investigate the potential mediating effects of PMC and health-related physical fitness on the relationship between AMC and MVPA across middle and late childhood. To investigate the potential differences in these mediating effects between middle and late childhood, multiple-group SEM analyses were conducted. Between-group differences were assessed for statistical significance using the Chi-square difference test. Model fit was evaluated using multiple indices: χ^2^ statistics (*p* > 0.05 indicating acceptable fit), the comparative fit index (CFI > 0.90), the Tucker-Lewis index (TLI > 0.90), the root mean square error of approximation (RMSEA < 0.08), and the standardized root mean square residual (SRMR < 0.08) (Kenny, 2020). All statistical analyses were conducted using the lavaan package in R software (Rosseel, 2012). Statistical significance was defined as *p* < 0.05.

## Results

### Descriptive characteristics of participants

A total of 578 children participated in the study, including 281 males (48.6%) and 297 females (51.4%). Among them, 273 (47.2%) were in middle childhood, and 305 (52.8%) were in late childhood. As summarized in Table 1, participants in the late childhood group demonstrated significantly higher levels of AMC, ball skills, locomotor skills, health-related physical fitness, BMI, vital capacity, and 1-min rope-skipping performance compared to the middle childhood group (all *p* < 0.001). They also exhibited faster sprint times in the 50-m dash (*p* < 0.001) and reported slightly higher PMC (*p* = 0.014). In contrast, the middle childhood group performed significantly better on the sit-and-reach test (*p* < 0.001). No significant difference emerged between the two age groups in daily MVPA levels (*p* = 0.438).

**TABLE 1:** Characteristics of the participants.

Variables	All (*n* = 578)	Middle childhood (*n* = 273)	Late childhood (*n* = 305)	*P*-value
BMI (kg/m^2^, M ± SD)	17.36 (2.71)	16.71 (2.09)	18.16 (3.14)	<0.001
Vital capacity (milliliters)	1,558 (493)	1,338 (372)	1806 (496)	<0.001
50-m dash (s)	10.6 (1.1)	11.0 (1.1)	10.3 (0.9)	<0.001
Sit-and-reach test (cm)	7.5 (5.5)	8.4 (5.1)	6.5 (5.7)	<0.001
1-min rope-skipping (number)	148 (36)	139 (35)	158 (34)	<0.001
Locomotor skills (score)	33.83 (5.50)	32.80 (6.85)	35.08 (2.67)	<0.001
Ball skills (score)	35.74 (6.89)	33.33 (7.64)	38.67 (4.34)	<0.001
AMC (score)	69.57 (10.50)	66.14 (12.32)	73.75 (5.29)	<0.001
PMC (score)	18.21 (4.08)	17.81 (4.31)	18.71 (3.74)	0.014
Health-related physical fitness (score)	82.23 (5.60)	81.23 (6.26)	83.23 (4.45)	<0.001
MVPA min/day	48.97 (16.30)	48.45 (17.12)	49.59 (15.26)	0.438

AMC, actual motor competence; PMC, perceived motor competence; MVPA, moderate-to-vigorous physical activity.

### Correlations among study variables

The bivariate correlations among AMC, PMA, health-related physical fitness, and MVPA for middle and late childhood are presented in Table 2. Correlation coefficients indicated that AMC was moderately correlated with PMC (*r* = 0.45) and highly correlated with health-related physical fitness (*r* = 0.53) in middle childhood. In late childhood, AMC was highly correlated with PMC (*r* = 0.78) but not with health-related physical fitness (*r* = 0.08). Additionally, AMC was highly correlated with MVPA in both middle (*r* = 0.61) and late childhood (*r* = 0.71). However, PMC was weakly correlated with MVPA in middle childhood (*r* = 0.24) and highly correlated in late childhood (*r* = 0.55). Finally, health-related physical fitness was moderately correlated with MVPA in middle childhood (*r* = 0.44) but not in late childhood (*r* = 0.10).

**TABLE 2:** Bivariate correlations among study variables for middle childhood (above diagonal) and late childhood (below diagonal).

Variable	BMI	VC	50 m	SR	RS	LOC	BALL	AMC	PMC	HRPF	MVPA
BMI	–	0.27^**^	−0.14*	−0.07	0.12*	0.14*	0.20^**^	0.20^**^	0.16^**^	0.22*	0.18^**^
Vital capacity (VC)	0.35^**^	–	0.29^**^	0.01	0.14*	0.20^**^	0.10	0.17^**^	0.09	0.40^**^	0.30^**^
50 m dash	0.03	0.01	–	0.05	0.31^**^	0.22^**^	0.32^**^	0.32^**^	0.27^**^	0.47^**^	0.32^**^
Sit and reach (SR)	0.10	0.03	0.09	–	0.02	0.01	0.15*	0.09	0.09	0.22^**^	0.12
Rope skip (RS)	0.14*	0.02	0.04	0.25^**^	–	0.37^**^	0.34^**^	0.42^**^	0.37^**^	0.64^**^	0.25^**^
Locomotor (LOC)	0.02	0.25^**^	0.02	0.19	0.04	–	0.45^**^	0.83^**^	0.52^**^	0.49^**^	0.54^**^
Ball skills (BALL)	0.02	0.06	0.00	0.15	0.06	0.09	–	0.87^**^	0.39^**^	0.42^**^	0.50^**^
AMC	0.01	0.17*	0.01	0.22	0.07	0.58^**^	0.86^**^	–	0.45^**^	0.53^**^	0.61^**^
PMC	0.01	0.08	0.01	0.05	0.17^**^	0.81^**^	0.73^**^	0.78^**^	–	0.50^**^	0.24^**^
HRPF	0.51*	0.66^**^	0.54*	0.55^**^	0.62^**^	0.09	0.05	0.08	0.31^**^	–	0.44^**^
MVPA	0.15*	0.05	0.06	0.16	0.13	0.19^**^	0.26^**^	0.71^**^	0.55^**^	0.10	–

AMC, actual motor competence; PMC, perceived motor competence; MVPA, moderate-to-vigorous physical activity; HRPF, Health-Related Physical Fitness. Middle childhood correlations are displayed above the diagonal (shaded); late childhood correlations are displayed below the diagonal.

^*^P < 0.05.

^**^P < 0.01.

### Structural equation modeling (SEM)

**FIGURE 1:**
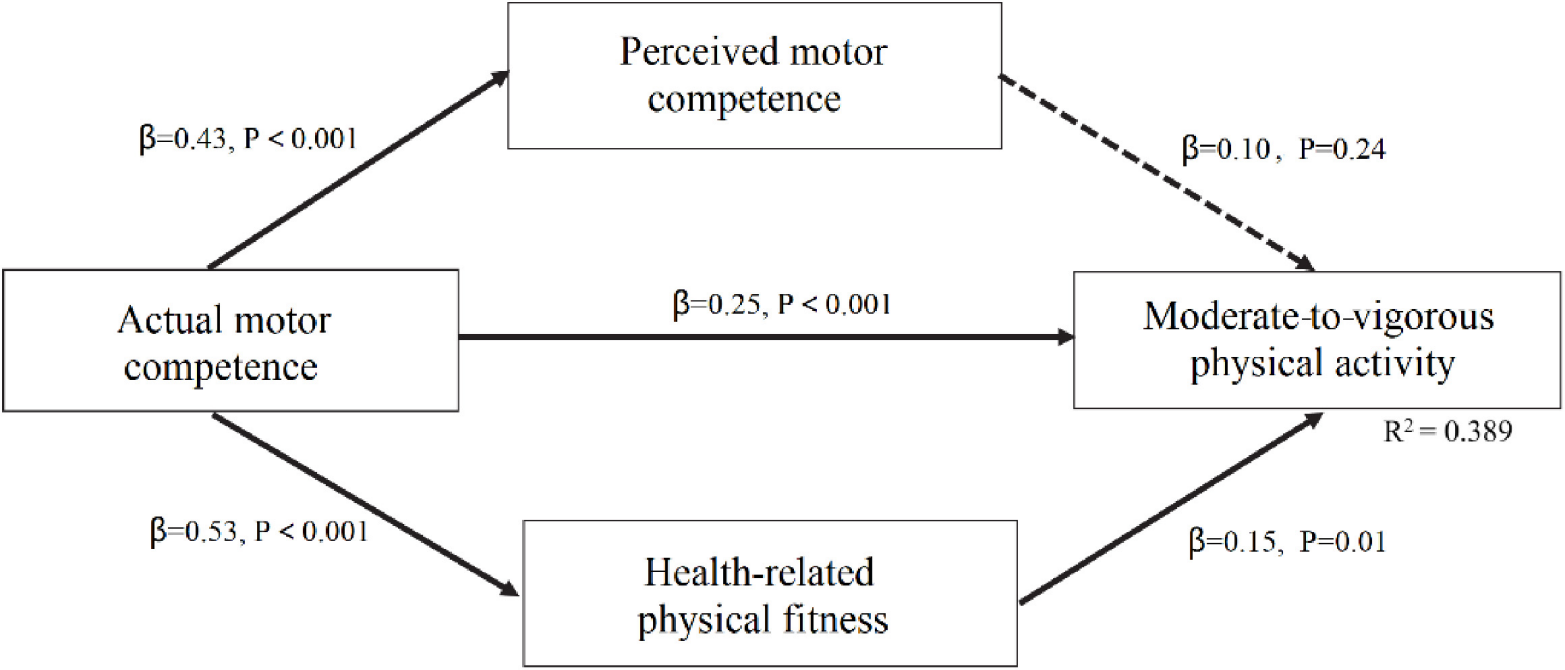
Mediating effects of perceived motor competence and health-related physical fitness on the association between actual motor competence and physical activity in middle childhood.

Structural equation modeling was used to analyze the mediating effect of PMC and health-related physical fitness in the relationship between AMC and MVPA in middle and late childhood. For middle childhood, the model showed a good fit to the data (χ^2^ = 5.527, *P* = 0.09; CFI = 0.991; TLI = 0.947; RMSEA = 0.079; SRMR = 0.023). As shown in Figure 1, the model demonstrated four direct paths: (a) from AMC to PMC (β = 0.43, *p* < 0.001); (b) from AMC to health-related physical fitness (β = 0.53, *p* < 0.001); (c) from AMC to MVPA (β = 0.25, *p* < 0.001); and (d) from health-related physical fitness to MVPA (β = 0.15, *p* = 0.01). The model also indicated significant indirect paths from AMC through PMC to MVPA (β = 0.04, *p* < 0.001) and from AMC through health-related physical fitness to MVPA (β = 0.08, *p* < 0.001). The total model explained 38.9% of the variance in MVPA for middle childhood.

**FIGURE 2:**
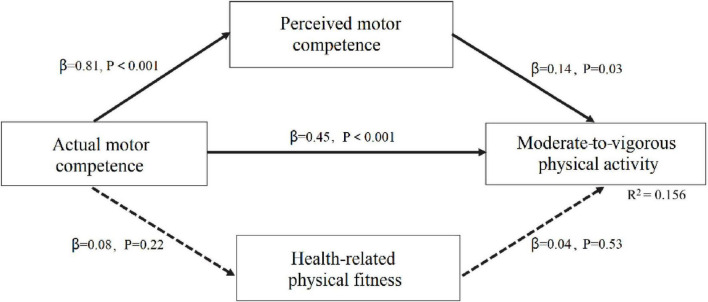
Mediating effects of perceived motor competence and health-related physical fitness on the association between actual motor competence and physical activity in late childhood.

For late childhood, the model fit parameters were satisfactory (χ^2^ = 22.652, *P* = 0.07; CFI = 0.966; TLI = 0.971; RMSEA = 0.061; SRMR = 0.049). As shown in Figure 2, the model exhibited three direct paths: (a) from AMC to PMC (β = 0.81, *p* < 0.001); (b) from AMC to MVPA (β = 0.45, *p* < 0.001); and (c) from PMC to MVPA (β = 0.14, *p* = 0.03). Additionally, the model revealed a significant indirect path from AMC through PMC to MVPA (β = 0.11, *p* < 0.001). However, the indirect paths from AMC through health-related physical fitness to MVPA were not significant (β = 0.003, *p* = 0.53). The model explained 15.6% of the variance in MVPA in late childhood.

The multiple-group SEM analysis revealed a significant difference in the mediating effects of PMC and health-related physical fitness between middle and late childhood, as indicated by a chi-square difference test (Δχ^2^ = 10.09, *p* < 0.05). Specifically, the mediating effect of PMC on the AMC-MVPA pathway was stronger in late childhood (β = 0.11) than in middle childhood (β = 0.04). In contrast, the mediating effect through health-related physical fitness diminished from middle childhood (β = 0.08) to late childhood (β = 0.003).

## Discussion

The aim of this study was to examine the mediating effects of PMC and health-related physical fitness on the relationship between AMC and PA in middle and late childhood. Two separate models were developed to explore the pathways from AMC through PMC and health-related physical fitness to MVPA. The results showed that both models demonstrated satisfactory fit, providing preliminary support for the hypothesized relationships among the variables in the model.

This analysis elucidates observed age-related differences that suggest a key potential developmental nuance characterizing the interplay between AMC, PMC, and MVPA. Although significant direct pathways were observed across both age cohorts, the magnitude of these relationships showed a marked increase from middle to late childhood. To illustrate, the association between AMC and PMC exhibited more than a 2-fold strengthening (β = 0.43 vs. β = 0.81), while the direct pathway from AMC to MVPA also demonstrated a considerable reinforcement (β = 0.25 vs. β = 0.45). This trend of AMC emerging as a progressively stronger correlate of perceptual and behavioral outcomes is consistent with multiple developmental perspectives. However, the differing PMC instruments across age groups require that this interpretation be made cautiously. Initially, with advancing cognitive maturation, children may develop a more refined capacity to assess their own competencies relative to peers (Robinson et al., 2015; de Witte et al., 2022). Consequently, older children might transition from a generalized optimism about their capabilities to a more realistic and nuanced self-concept that is critically shaped by their actual motor competencies. Furthermore, MVPA for older children typically involves more complex, structured, and skill-oriented activities, such as team sports. Within these contexts, robust AMC serves not merely as an advantage but as a fundamental prerequisite for consistent and successful engagement. These findings are consistent with aspects of the conceptual model proposed by Stodden et al. (2008), which posits that the strength of association between AMC, PMC and MVPA varies with age. In late childhood, a direct association was observed PMC and MVPA; however, this association was not found in middle childhood. This pattern suggests that as children grow older, those with higher PMC tend to be more physically active. Interpreting this observed developmental trajectory is complicated by the use of divergent PMC measures across different age groups. For younger children, the PSPCSA gauged perceived physical competence within broad, everyday contexts like climbing. In contrast, the SPPC, used with older children, measured perceived athletic competence in more narrowly defined, sport-oriented settings. This methodological discrepancy raises a key question: do the stronger correlations seen in late childhood indicate a genuine developmental shift—for instance, a consolidation of psycho-behavioral pathways due to cognitive maturation—or are they merely an artifact of the assessment tools? The inherent alignment between the SPPC’s focus on “athletic competence” and the activities quantified by MVPA metrics further complicates the isolation of true developmental effects from those influenced by the construct’s measurement (Gallahue and Ozmun, 2006). Therefore, the age-group differences reported here should be interpreted as reflecting a combination of potential developmental change and measurement variance.

In addition, the data indicated that in middle childhood, AMC was directly related to health-related physical fitness, and the health-related physical fitness was directly related to MVPA. In other words, children with better AMC tended to have higher health-related physical fitness, which further promoted their PA levels. In contrast, no direct relationships were found between AMC and health-related physical fitness, or between health-related physical fitness and MVPA, in late childhood. These findings were inconsistent with the conceptual model proposed by Stodden et al. (2008), which proposed that in late childhood and adolescence, higher levels of AMC should be strongly associated with greater health-related physical fitness and PA levels. One potential explanation for these inconsistencies is the Chinese educational system. Rooted in Confucian principles, Chinese culture places a strong emphasis on academic achievement, often prioritizing exam performance over physical development (Johns and Vertinsky, 2006). Empirical evidence substantiates this priority: students in this cohort dedicate 13.8 h per week to extracurricular learning, nearly twice the international benchmark (OECD, 2017). This rigorous academic schedule directly displaces time available for PA, a limitation that becomes particularly pronounced for old children facing highly competitive entrance examinations. A further consideration is the composition of the health-related physical fitness variable. Although using a composite score is common, this approach may mask the distinct mediating influence of individual fitness components. Cardiorespiratory fitness, for example, can be theorized to have a more direct and robust relationship with MVPA than other elements, such as muscular strength (Chen et al., 2018). Consequently, future studies should investigate both overarching and discrete fitness measures to achieve a more refined analysis. The present findings are further corroborated by Barnett et al.’s (2022) systematic review, which designated the evidence for the fitness-mediated pathway as “strongly positive.” This conclusion stands in stark contrast to the absence of this pathway in the late childhood data from the current study, which—when viewed alongside Barnett et al.’s (2022) “indeterminate” rating for the longitudinal AMC-PA link—suggests that these developmental mechanisms are more dynamic and culturally contingent than originally theorized. This convergence of evidence underscores the necessity for longitudinal research that can elucidate how and when contextual factors, like academic pressure, alter these critical developmental pathways.

This study also identified significant indirect pathways among the variables. Notably, a key finding was the presence of an indirect pathway from AMC through PMC to MVPA in both middle and late childhood, as well as from AMC through health-related physical fitness to MVPA in middle childhood. These findings align with those of Khodaverdi et al. (2016), Gu et al. (2021) for middle childhood, and Gu et al. (2017) for late childhood. Furthermore, the study revealed that the mediating effect of PMC on the AMC-MVPA pathway was stronger in late childhood than in middle childhood. To the best of current knowledge, this study represents the first empirical investigation into the differences in indirect associations among these variables across middle and late childhood. The findings show a pattern that aligns with the conceptual model proposed by Stodden et al. (2008). If the observed differences reflect true development, as children’s cognitive abilities develop from middle to late childhood, older children can more accurately estimate their motor competence compared to younger children. This is supported by various research samples, such as Barnett et al. (2015) for 4–8-year-old, Raudsepp and Liblik (2002) for 10–13-year-old, and de Witte et al. (2022) for 6–12-year-old, which consistently found stronger correlations between AMC and PMC in older age groups. Higher AMC enhances children’s PMC, making them more likely to persist in PA and thereby achieve higher activity levels in late childhood (Cattuzzo et al., 2016).

After controlling for child gender, parental education level, and family SES, the model explained 38.9% of the variance in MVPA during middle childhood. However, by late childhood, the model’s explanatory power decreased to 15.6%. These results suggest that while AMC, PMC, and health-related physical fitness play a significant role in predicting MVPA in middle childhood, their influence diminishes in late childhood. This observed attenuation can be understood through an alternative theoretical perspective proposed by Zi and De Geus (2025), which posits that cross-sectional correlations between motor competence and PA may not reflect direct causation but could be substantially confounded by shared genetic predispositions and familial environment. The present findings offer compelling empirical support for this proposition. The robust, fitness-mediated relationship evident in middle childhood may be largely attributable to these common underlying familial factors. As children mature into late childhood and develop greater autonomy, the influence of the familial milieu diminishes, giving way to the increased salience of peer networks, academic pressures, and evolving self-concept (Klazine et al., 2007). This progressive dilution of the shared familial context logically leads to a weakened statistical association between MC and MVPA—a pattern that aligns precisely with the documented results. Consequently, the developmental trajectory observed in this study may signify not only a shift in psychosocial mediators but also the receding influence of the familial confounders that initially co-determined both motor competence and activity behaviors.

The strengths of this study include the use of a comprehensive, process-oriented battery to assess children’s AMC and objective techniques (e.g., accelerometers) to evaluate children’s PA and health-related physical fitness levels. These methods provide greater accuracy and objectivity in determining these variables. Additionally, confounding variables were controlled in all analyses. Despite these strengths, several limitations should be acknowledged. First, as with all cross-sectional studies, the data were collected at a single time point, making it impossible to establish causal relationships. Future research could employ longitudinal or experimental designs to better understand the interaction among variables. Second, this study used SEM to analyze the relationships between variables. Given that the model may be influenced by potential multicollinearity, caution is warranted when interpreting the preliminary findings (Vatcheva et al., 2016). A third, significant limitation pertains to the assessment of PMC. The age-appropriate instruments that were utilized, while valid for their respective stages, measured subtly different constructs. These ranged from a global sense of physical capability in younger children to a more narrow focus on athletic prowess in later childhood. As a result of this methodological inconsistency, one cannot ascertain with certainty if the outcomes reflect an authentic developmental progression or are merely a byproduct of the instruments used. Therefore, this study underscores the urgent necessity for creating and validating a cohesive PMC scale, designed to be developmentally sensitive and applicable throughout the various stages of childhood.

## Conclusion

The present study demonstrates a positive association between AMC and PMC, AMC and MVPA, and AMC and health-related physical fitness during middle childhood. Similarly, in late childhood, direct association were identified between AMC and PMC, as well as AMC and MVPA. Furthermore, two indirect pathways were found in middle childhood: from AMC through PMC to MVPA, and from AMC through health-related physical fitness to MVPA. In contrast, only one indirect pathway—from AMC through PMC to MVPA—was identified in late childhood. Collectively, these results highlight the imperative for interventions that are sensitive to both developmental stage and socio-cultural context. Middle childhood presents a critical period for PA promotion by building upon the synergistic development of AMC, PMC, and health-related physical fitness. To achieve a more definitive understanding, the field must progress from documenting cross-sectional associations to employing methodologically robust longitudinal and genetically informed designs (e.g., twin or family studies), integrated with controlled trials. Such methodologies are indispensable for disentangling true causal pathways from shared familial confounding and, ultimately, for informing more potent and precisely targeted strategies to enhance PA throughout childhood.

## Data Availability

The raw data supporting the conclusions of this article will be made available by the authors, without undue reservation.
